# Effect of Additives on the *in situ* Laccase-Catalyzed Polymerization of Aniline Onto Bacterial Cellulose

**DOI:** 10.3389/fbioe.2019.00264

**Published:** 2019-10-17

**Authors:** Euijin Shim, Jennifer Noro, Artur Cavaco-Paulo, Carla Silva, Hye Rim Kim

**Affiliations:** ^1^Department of Clothing and Textiles, Sookmyung Women's University, Seoul, South Korea; ^2^International Joint Research Laboratory for Textile and Fiber Bioprocesses, Jiangnan University, Wuxi, China; ^3^Centre of Biological Engineering, University of Minho, Campus of Gualtar, Braga, Portugal

**Keywords:** aniline, conductivity, additives, oxidation, laccase

## Abstract

Laccase-mediated systems are a green route to accelerate the oxidation of aniline and obtain polyaniline with conductive properties. The synthesis of green polyaniline (emeraldine salt) was herein improved by the inclusion of additives like sodium bis (2–ethyl hexyl) sulfosuccinate (AOT) and potassium hexacyanoferrate (II) (KHCF) in the medium. The aniline polymerization was confirmed by the detection of the absorption band typical of emeraldine salt at 420 nm, typical of the semiquinoid radical cation, and of the polaron absorption band at 700–800 nm, corresponding to the distinctive signal of doped or partial doped aniline. The oligomers and/or polymers obtained were characterized by spectrometry techniques, namely ^1^H NMR and MALDI-TOF, and the bacterial cellulose (BC) conductivity was assessed by means of a four-point probe electrical conductivity technique. The best polymerization results were obtained with 5 mM AOT, 10 mM KHCF, and 25 U/mL of laccase. The synergistic effect between both additives in the presence of a catalyst leads to obtaining BC samples coated with green polyaniline with promising electric conductive properties.

## Introduction

Bacterial cellulose is a versatile biopolymer produced by *Acetobacter xylinum* with exceptional properties, like high purity, high porosity, high permeability to liquid and gases, high water-uptake capacity, tensile strength, and an ultrafine network. Due to its excellent properties, this material can find applications in several fields including food, cosmetics, biomedicine, and drug delivery (Lin et al., 2014; Wang et al., 2016; Yue et al., 2016).

Regarding specific applications, bacterial cellulose has been explored as a template for the *in situ* polymerization of a panoply of compounds from phenols to polyamines. An example has been reported by Song et al. who studied the *in situ* polymerization of several flavonoids by laccase onto a bacterial cellulose support (Song et al., 2018). The usage of bacterial cellulose as a template for aniline polymerization has also been undertaken. Bacterial cellulose composites have been produced by a combination of several polymeric matrices or by coating *in situ* polymerized polymers onto bacterial cellulose nano fibers (Alonso et al., 2018; Shim et al., 2019). Lee et al. studied the preparation of bacterial cellulose/polyaniline composite films by chemical oxidative polymerization of aniline (Lee et al., 2012). Aiming to enhance the electrical conductivity of bacterial cellulose, this material was coated with conductive polymers (Xu et al., 2013; Jasim et al., 2017). Different oxidizing agents have also been explored on the production of polyaniline coated bacterial cellulose membranes, nanofibers, and hydrogels (Müller et al., 2012; Shi et al., 2012; Marins et al., 2014). Conducting bacterial cellulose can find multiple applications in electronic devices, namely transistors, displays, sensors, energy-storage, and memory cells, in polymer nanolithography, inhibition of corrosion, catalysis, and medicine, among others (Sapurina and Shishov, 2012). Its high stability and unique properties, made polyaniline (PANI) the first, among conducting polymers, to be used as an antistatic coating electrode material for batteries and condensers, or as a corrosion inhibitor and detecting material for sensors (Sapurina and Shishov, 2012). In general, PANI possesses conductivity values within the 10^−10^-10^1^ S·cm^−1^ range; the chains of conducting PANI have an ordered structure containing regularly alternating phenyl rings and nitrogen-containing groups (Sapurina and Shishov, 2012). The final polymer is non-toxic, stable in harsh chemical environments, has high thermal stability and low costs are associated to its production.

Despite all the efforts at elucidating the mechanism of aniline oxidation, some features are still unclear, namely the high selectivity of the mechanism, the influence of the pH and the influence of different types of additives.

The main aim of this work is to evaluate the role of additives, namely a surfactant and a radical initiator, on the enzymatic-assisted polymerization of aniline to confer BC a conductive character. The novelty of the study relies on the use of additives to increment the enzymatic oxidation of aniline using laccase as a catalyst. Potassium hexacyanoferrate (II) trihydrate (KHCF) was chosen as a radical initiator and sodium bis (2–ethyl hexyl) sulfosuccinate (AOT) as the surfactant, and their role on the oxidation of aniline was evaluated and compared. The addition of KHCF, already described as being the surfactant used for the polymerization of aniline in the presence of laccase, is to be highlighted. The *in situ* polymerization of aniline onto BC was conducted using laccase as a catalyst. The PANI produced was characterized by ^1^H NMR and MALDI-TOF spectrometry and the coating of bacterial cellulose with the conductive PANI was monitored by SEM, FTIR, TGA, DSC, and XRD analysis. The electrical conductivity of coated bacterial cellulose samples was assessed by using the four-point probe method.

## Experimental

### Materials

For the production of bacterial cellulose (BC), glucose (Duksan Pure Chemicals Co., Seoul, Korea) was used as carbon source and a mixture of yeast extract (Becton, Dickinson and Company, Sparks, USA) and peptone (Becton, Dickinson and company, Sparks, USA) were used as nitrogen sources. The following chemicals were used without further purification: acetic acid (Duksan Pure Chemicals Co., Seoul, Korea), sodium acetate (Sigma, Saint Louis, USA), hydrogen peroxide (Duksan Pure Chemicals., Korea), aniline (Junsei Chemicals Co., Tokyo, Japan), potassium hexacyanoferrate (II) (Fisher Scientific, Loughborough, UK), Bis(2-ethylhexyl) sulfosuccinate sodium salt (Tokyo chemical industry Co., Tokyo, Japan). Citric acid monohydrate (Sigma, Saint Louis, USA), sodium phosphate dibasic dehydrate (Riedelde Haën, Seelze, Germany). Laccase (EC 1.10.3.2.) from *Myceliophthora thermophila* (MtL) was supplied by Novozymes (Denmark).

### Production of Bacterial Cellulose (BC)

BC samples were produced and pretreated according to the previously methodology reported (Han et al., 2019). The Hestrin-Schramm (HS) medium was prepared adding glucose as a carbon source (20 g/L), with a mixture of yeast extract and peptone powder as the nitrogen source (5 g/L each). Both were added to distilled water and mixed until dissolution. Afterwards the solution was boiled at 100°C for 10 min. BC was cultured statically at 26°C for 8 days. The BC samples were washed and bleached according to the method previously reported (Han et al., 2019).

### “*In situ”* Laccase-Assisted Polymerization of PANI Onto BC

**Scheme 1:**
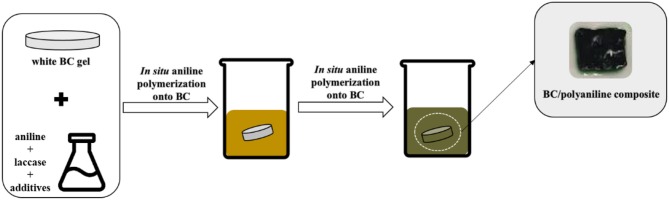
*In situ* laccase-assisted polymerization of aniline onto BC samples.

The BC samples were cut into small pieces (1.5 cm^2^) and placed in a 100 mL flask containing a mixture of 25 U/ml of laccase (634 U/mL), 5 mM of AOT, 10 mM of KHCF and 50 mM of aniline, in a final volume of 20 mL of acetate buffer (pH 4). The samples and solutions were stirred for 24 h at room temperature in a water bath (Grant, United Kingdom). At the end of each experiment, the BC samples were passed in a filter paper and washed thoroughly with distilled water to remove the by-products and the remaining starting reagents. Afterwards the BC samples coated with PANI were dried in a convection oven (OF-21, Jeio tech Co.) at 35°C for 24 h (Scheme 1).

### Characterization of PANI

#### UV/Visible Spectroscopy

The polymerization of aniline was followed by UV-Visible spectroscopy using a 96-quartz microplate reader (SynergyMx, Shimadzu, Japan) under the wavelength range of 230–700 nm.

#### ^1^H NMR and MALDI-TOF Spectroscopy

The ^1^H NMR spectroscopy of PANI was assessed using a Bruker Avance 400 (400 MHz). CDCl_3_ was used as deuterated solvent, using the peak solvent as internal reference. The oligomer/polymer products obtained were also characterized by MALDI-TOF mass spectrometry using a Bruker Autoflex Speed instrument (Bruker Daltonics GmbH) equipped with a 337 nm nitrogen laser. The matrix solution for MALDI-TOF measurements was prepared by dissolving a saturated solution of 2,5-dihydroxybenzonic acid (DHB) in TA30 solution. Samples were spotted onto a ground steel target plate (Bruker part *n*° 209519) and analyzed in the linear negative mode by using factory-configured instrument parameters suitable for a 0.4–4 kDa m/z range (ion source 1: 19.5 kV; ion source 2: 18.3 kV). The time delay between laser pulse and ion extraction was set to 130 ns, and the laser frequency was 25 Hz. The *M*_*n*_ (number-average molecular weight) and *M*_*w*_ (weight-average molecular weight) of PANI obtained after oxidation was obtained by MALDI-TOF direct analysis and according to the equations:

(1) $$M_{n\;}=\;\frac{\sum n_{i}\;M_{i}}{\sum n_{i}}$$

(2) $$M_{w\;}=\;\frac{\sum n_{i}\;M_{i}^{2}}{\sum n_{i}\;M_{i}}$$

Where *n*_*i*_ is the relative abundance of each peak; *M*_*i*_ is the *m/z* correspondent to each peak (Su et al., 2018).

#### Scanning Electron Microscopy

The powder samples were characterized using a desktop scanning electron microscope (SEM) coupled with energy-dispersive X-ray spectroscopy (EDS) analysis [Phenom ProX with EDS detector (Phenom-World BV, Netherlands)]. All results were acquired using the ProSuite software integrated with Phenom Element Identification software, allowed for the quantification of the concentration of the elements present in the samples, expressed in either weight or atomic concentration. The powder samples were added to aluminum pin stubs with electrically conductive carbon adhesive tape (PELCO Tabs™). Samples were coated with 2 nm of Au (20 Angstrom) for improved conductivity. The aluminum pin stub was then placed inside a Phenom Standard Sample Holder (SH), and different points were analyzed for elemental composition. EDS analysis was conducted at 15 kV with intensity map.

### Characterization of BC/PANI Composites

#### FTIR-ATR

To analyze the chemical structure of BC coated with PANI under different conditions, FTIR-ATR spectra were conducted using an ATR FTIR, IRAffinity-1S (SHIMADZU). Scans were completed between 4,000 and 400 cm^−1^ at a resolution of 8 cm^−1^, using 45 scans. Baselines for each sample spectrum were normalized using spectrum software.

#### X-ray Diffraction

The changes on the surface structure of BC samples when coated with PANI were investigated by X-ray diffraction (XRD) using a New D8-ADVANCE (multi-purpose diffractometer, Bruker-AXS, Fitchburg, USA). XRD patterns were recorded at the CuKa radiation wavelength of 1.54180 A° and generated at a voltage of 40 kV and a filament emission of 40 mA. Samples were scanned in the range from 5 to 40 degrees at a scan speed of 1/min. The crystallinity index (CI) was calculated from the height ratio between the intensity of the crystalline peak (I_002_-I_AM_) and total intensity (I_002_) after subtraction of the background signal measured without BC, following the equation:

(3) $$\begin{array}{r} CI=\;\frac{I_{002}\;-\;I_{am}}{I_{002}} \end{array}$$

#### Conductivity of Coated BC Samples

Electrical conductivity of coated BC samples was measured with a CMT-series (Changmin Tech Co., Ltd) using a four-point probe technique placing them under a pre-defined distance between. The conductivity was calculated according to the following equation:

(4) $$\begin{array}{r} Conductivity\left(\sigma \right)=1/\rho \left(S.m^{-1}\right), \end{array}$$

Resistivity can be calculated with ρ = 2πS(V/I), where *S* is the probe spacing (mm), which was kept constant, *I* is the supplied current in microamperes, and the *V* is corresponding voltage measured in millivolts. Electrical conductivity can be computed using σ1 = 1/ρ (Yue et al., 2016).

#### Color Evaluation of Coated BC Samples

The color (H), brightness (V), and saturation (C) of the polymerized BC were determined using Munsell's colorimetric transformation method, by measuring the X, Y, and Z values of the sample using a computational color-matching (CCM) system (JX-777, Japan). The color data for the dried samples was determined using a CCM system and illuminant D_65_ with a 10° standard observer. The color strength (K/S) was calculated from the reflectance values using the Kubelka–Munk equation:

(5) $$\begin{array}{r} K/S={\left(1-R\right)}^{2}/2R \end{array}$$

where *R* is the reflectance, expressed as a proportional value; *K* is the absorption coefficient; and *S* is the light-scattering coefficient. The *K/S* values were presented as checksum *K/S*. All measurements were performed at least 10 times.

#### Morphological Characterization—Scanning Electron Microscopy

The BC samples were characterized using a desktop scanning electron microscope (SEM) [Phenom ProX (Phenom-World BV, Netherlands)]. All results were acquired using the ProSuite software. The samples were added to aluminum pin stubs with electrically conductive carbon adhesive tape (PELCO Tabs™), with the excess removed using compressed air. Samples were coated with 2 nm of Au for improved conductivity. The aluminum pin stub was then placed inside a Phenom Standard Sample Holder, analysis was conducted at 10 kV with intensity image.

## Results and Discussion

### Enzymatic Polymerization of Aniline

The oxidation of aniline by laccase at pH = 4 is favored when using an enzyme with high-redox potential. Despite the low redox-potential of Mtl, this catalyst was chosen for the oxidation and the experiments were conducted for longer periods of time than that it would be necessary if a high-redox potential was used.

**Figure 1:**
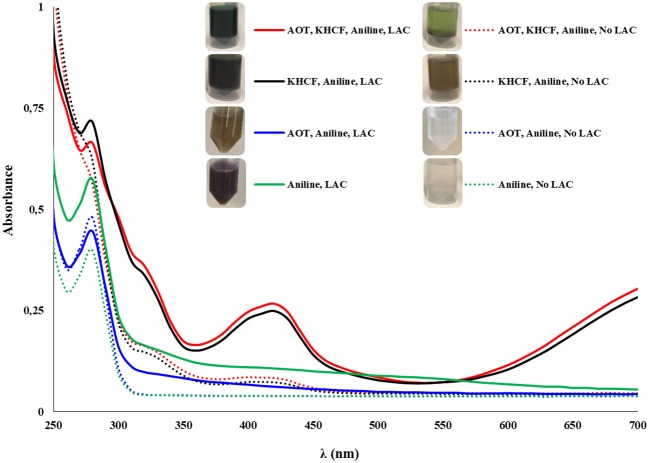
UV-visible spectra of PANI synthesized after 24 h of reaction with MtL (25 U/mL); the experiments were performed in the presence of an anionic surfactant as template (AOT) and/or a radical initiator (KHCF) (5 mM of AOT, 10 mM of KHCF, and 50 mM of aniline).

The synthesis of PANI with electro-conductive capacity (emeraldine salt) was conducted using 50 mM of acetate buffer (pH 4) as a doping agent to keep the aniline monomer protonated. We examined different media containing an anionic surfactant AOT and a radical initiator, KHCF, to function as (i) a template facilitating the *p*-directed coupling of the monomers, (ii) as an anionic dopant to obtain conductive PANI, and (iii) to improve the polymer solubility in water by micelles aggregation. The electronic spectrum (Figure 1) shows that after oxidation reaction with laccase (25 U/mL), the presence of a template together with a radical initiator settle the product's properties concerning color, polymerization degree, structure, and electrical properties. Green PANI was achieved when using KHCF alone or together with AOT. The UV-visible spectra of the green PANI synthesized using both additives revealed the absorption bands typical of emeraldine salt at 420 nm, typical of the semiquinoid radical cation (Zhang et al., 2011) and, at 800 nm, the characteristic signal of doped or partial doped PANI resulting from π-polaron electronic transitions (Junker et al., 2014). Due to equipment constraints it is not possible to present the complete spectra evidencing the complete band at 750 nm, however, from Figure 1 the appearance of this band is clear, especially on samples oxidized using both AOT and KHCF additives, confirming the complete or partial doping. This behavior was previously observed by De Salas et al. (2016) when they studied the role of different surfactants and templates for the aniline polymerization by laccase. The spectra of the fractions oxidized in the presence of AOT and laccase did not display the typical peak of emeraldine salt at 420 nm nor at 750 nm, as it would be expected regarding the results of MALDI-TOF presented further. Looking to the pictures of the solution, it is clear the lack of solubility of the oligomers/polymers, which due to the collapse of micelles resulted in dark-colored precipitates hindering the accurate spectral evaluation. The oxidized aniline was, however, detected by mass spectroscopy analysis. Also noteworthy is the formation of green polymer in the absence of laccase, even when KHCF is used alone or in combination with AOT. We may assume that the polymer was formed by inducing [Fe(CN)_6_]^3−^ ions from KHCF during oxidative polymerization. [Fe(CN)_6_]^3−^ ions were reduced into [Fe(CN)_6_]^4−^ ions which were reoxidized back into [Fe(CN)_6_]^3−^, by redox processes during laccase catalyzed polymerization (Ramanavicius et al., 2016). Moreover, AOT revealed high performance as a doping template giving rise also to green soluble conductive polymer, but in this case the presence of the catalyst is imperative to obtain aniline oxidation.

### Properties of Products of Aniline Oxidation

#### PANI Characterization by ^1^H NMR and MALDI-TOF

Previous works related with laccase/O_2_-catalyzed oxidation of aniline have been reporting mixtures of different products and not a single type of PANI-ES molecule with a fully defined chemical structure. During enzyme-assisted processing and the formation of aniline radicals, different follow-up reactions may occur and the insolubility of the products may hinder their complete structural evaluation. Herein, the PANI powders were characterized through ^1^H NMR and MALDI-TOF (Figures S1–S3) which confirmed the presence of oligomeric and polymeric species in the oxidized fractions obtained. From ^1^H NMR spectra one can observe the peaks of the substrate, aniline, between δ_H_ 6.68–7.21 ppm (Figures S1, S2). The signal multiplicity is observed as one triplet at δ_H_ 6.78 ppm, and two doublets of doublets (at δ_H_ 6.71 and at δ_H_ 7.18 ppm). Based on the signals between δ_H_ 6.7 and 6.8 ppm, we inferred the polymer conversion (Table 1). In all spectra, with the exception of (d) and (e), the aniline peak disappear completely, indicating the full conversion of the substrate into PANI. On the oxidation of aniline carried out only with laccase (spectrum d), a conversion of 58% was inferred, while on the reaction of oxidation of aniline using only AOT (spectrum e), a conversion of only 50% is observed.

**Table 1:** Characterization of the products of aniline polymerization by laccase in the presence of additives.

	**Calculated according MALDI-TOF data**			**Calculated according ^**1**^H NMR data**
	**M_**n**_**	**M_**w**_**	**PDI (M_**w**_/M_**n**_)**	**Conversion (%)**
Aniline^*^+ laccase	477	530	1.1	58
Aniline + KHCF^Δ^ + No laccase	593	633	1.1	100
Aniline + KHCF + laccase	645	767	1.2	100
Aniline + AOT^¥^+ No laccase	1092	1808	1.7	51
Aniline + AOT + laccase	876	1470	1.7	100
Aniline +AOT + KHCF + No laccase	702	1123	1.6	100
Aniline + AOT + KHCF + laccase	599	718	1.2	100

* *Aniline (Mw): 93.13 g/mol*.

Δ *KHCF (Mw): 422.39 g/mol*.

¥ *AOT (Mw): 444.56 g/mol*.

From the results obtained we may also verify that the polymer peaks appear between δ_H_ 7.1 and 7.6 ppm with a different pattern than in the starting material. It is also perceptible that, independently of the reactional components used, the oxidation give rise to oligomers/polymers with similar structure.

PANI is a polymer formed by the bonding of the amine group of aniline, with a carbon at the *para*-position of another aniline moiety. This bond is detected in the NMR spectra, given the signal multiplicity attained by the formed polymer. A set of doublets/doublet of doublets are observed, with coupling constants around *J* = 8 Hz (*ortho* coupling) and *J* = 1.6 Hz (*meta* coupling). These findings agree with the suggested structure of the polymer previously reported (Su et al., 2018).

The MALDI-TOF spectra of aniline oligomers obtained by laccase-mediator synthesis in the presence of additives (Figure S3) have intense peaks with m/z 365, 454, 595, 636, 724, 800, 912, 1,355, 1,798, 2,240, 2,595, and 3,038, corresponding to 4-33 dimensional fragments of PANI. Table 1 displays the characterization of the oligomers/polymers after oxidation and isolation, obtained by ^1^H NMR and MALDI-TOF analysis. The data confirms the occurrence of oligomers and polymers after aniline laccase-assisted polymerization since low and high molecular mass polymeric fractions were detected. We have found oligomerization degrees of up to 33 residues in the dark PANI obtained in the presence of AOT and up to 9 residues in the soluble green products obtained in the presence of KHCF (Table 1). The data also reveal that the additives used displayed differentiated roles as templates of aniline oxidation. As previously confirmed by De Salas et al. (2016), high molecular species are obtained in the presence of AOT templates, whereas smaller species are produced using KHCF. From the combination of both templates results similar oligomeric species as obtained with AOT alone. The presence of laccase is only significant when KHCF is applied as additive alone. When together with the AOT template, the laccase action is hindered by the micelles formed, and smaller species are obtained.

The MALDI-TOF and ^1^H NMR results also revealed that the oxidation conducted in the presence of BC did not interfered with the type of polymer nor with the amount of polymer formed. The difference relies on the amount of polymer that is recovered in the supernatant after polymerization which is higher when BC is not included in the system. For this reason we present herein only the characterization of the obtained polymers in the absence of the support.

#### PANI Morphology

**Figure 2:**
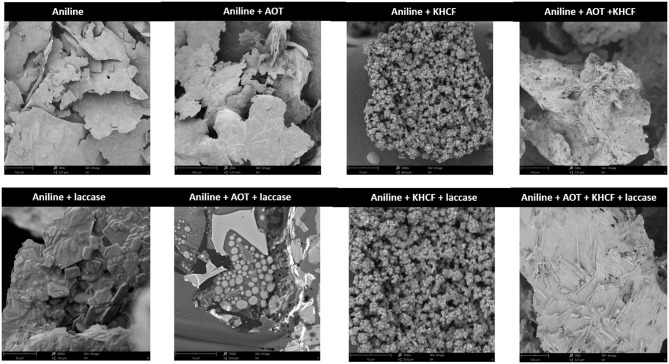
Scanning electron microscopy of products of aniline oxidation by laccase using additives.

It has been postulated that there is no direct correlation between conductivity and the structural properties of polymers, namely molecular mass and the type of supramolecular structure (Sapurina and Shishov, 2012). PANI morphologic structure is directly dependent on the external templates used for oxidation, oligomers may assume differentiated forms like microspheres or two-dimensional formations, and high molecular weight PANI may acquire one-dimensional (nanofibers or nanotubes) or three-dimensional structures (granules) (Sapurina and Shishov, 2012). We have investigated by SEM the final structure of the polymers synthesized and the results can be depicted in Figure 2. The oxidation of aniline only in presence of laccase, in absence of any template, gave a disordered crystal-like structure, attributed to the irregular branched polymerization of aniline. The oxidation in the presence of templates gave rise to differentiated structures. The polymerization of aniline in the presence of KCHF resulted in microspheric structures, corresponding to smaller species, whilst the oxidation in the presence of both templates resulted in structures similar to one dimension nanofibers and three dimensional granules. The resulting structures are in accordance with what has been described for small and bigger oxidized species, respectively. As depicted in Figure 2, the structure of PANI samples obtained in the presence of AOT alone was not easily visualized, probably due to the high micelles concentration which have constrained and uncovered the oligomeric/polymeric structures produced.

### Characterization of BC Coated With PANI

#### FTIR

The FTIR-ATR of BC and BC coated with PANI obtained through different reactional conditions are shown in Figure S4. The spectrum of BC reveal all the characteristic peaks of BC and thereby confirmed the basic structure of pure cellulose at 3,332 cm^−1^, characteristic peak for –OH stretching. The intensity of this peak is reduced when PANI is incorporated into the BC fibers (data not shown). The absorption spectra of BC coated with PANI did not show any N-H stretching absorption at 3,442 cm^−1^ as reported previously. We may assume that BC reacted via dehydration-condensation with the polymer, PANI, resulting in the reduction of the O-H peak and of the N-H bonds (Karim et al., 2005; Jasim et al., 2017). The coating of BC with PANI can be evidenced by the intensity reduction of the sharp band at 1,065 cm^−1^ (C–O–C stretching vibrations) and of the band at 2,920 cm^−1^ (not shown in the spectra selected), assigned to the aliphatic C–H stretching vibration (Wang et al., 2012).

**Figure 3:**
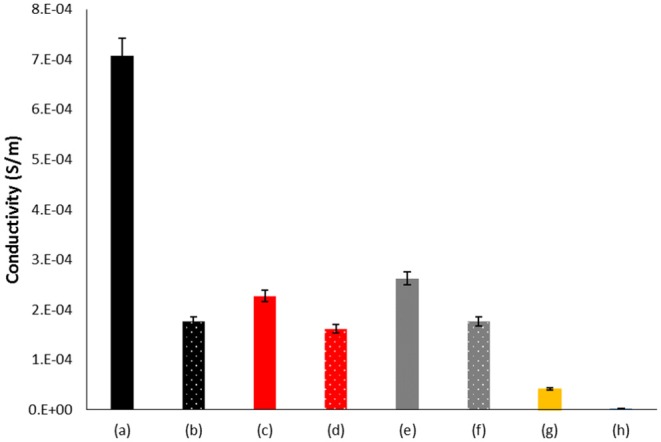
Electrical conductivity of BC coated with PANI after oxidation under different conditions; (a) BC + aniline + AOT+ KHCF + laccase; (b) BC + aniline + AOT + KHCF; (c) BC + aniline + KHCF + laccase; (d) BC + aniline + KHCF; (e) BC + aniline + AOT+ laccase +; (f) BC + aniline + AOT; (g) BC + aniline + laccase; (h) BC + aniline.

Usually, PANI exhibits an absorption band at 1,573–1,592 cm^−1^ and at 1,470 cm^−1^, corresponding to the stretching vibration of quinoid and benzoid structure, respectively (Müller et al., 2012; Mashkour et al., 2016). Considering the detection of these peaks also in the BC, any small change in this region of the spectra, related with PANI attachment to BC, is masked by the peaks of the control. Moreover, the characteristic band of PANI in coated BC clearly observed at 1,448 cm^−1^ is assigned to the C–C stretching of benzenoid rings (Figure 3; Wang et al., 2012) (Table S1).

#### XRD

A comparative XRD study (10–60°) between BC and BC coated with PANI is shown in Figure S5. The spectrum of BC displays two main diffraction peaks at 2θ = 14.5° and 23°, characteristic of cellulose type I (I_α_ and I_β_ polymorphs), which are assigned to the (110) and (220) diffraction planes. The spectrum of BC/PANI composites present peaks at 2θ = 35.2° related with PANI (Marins et al., 2011, 2014). As observed in Table 2, the crystallinity degree of BC/PANI prepared with KHCF and AOT by laccase catalysis was determined from X-ray diffraction as previously described (Marins et al., 2014). The crystallinity of conducting polymers corresponds to a more ordered system which can display a metallic-like conductive state (Rahy and Yang, 2008). The data obtained show that the coated BC displays higher crystallinity than BC control incremented by the presence of entrapped PANI (Marins et al., 2014). The measurements performed in several points of the samples, reveal a uniformity of the crystallinity which might be attributed to the interaction of the PANI with the -OH groups at the surface of BC, resulting in a uniform distribution of the polymer (Jasim et al., 2017).

**Table 2:** Cellulose crystallinity of BC/PANI samples.

	**Crystallinity (%)**
Bacterial cellulose (BC)	68.3
BC + aniline + laccase	72.1
BC + aniline + AOT + laccase	73.6
BC + aniline + AOT	70.5
BC + aniline + KHCF + laccase	73.0
BC + aniline + KHCF	70.9
BC + aniline + AOT + KHCF + laccase	76.1
BC + aniline + AOT + KHCF	72.2

#### Electrical Conductivity of BC Samples Coated With PANI

Figure 3 shows the conductivity of BC samples coated with aniline oxidized species. The highest conductivity is obtained for samples coated with PANI obtained by laccase-assisted oxidation in the presence of both additives, AOT and KHCF. This is an expected result since these conditions gave rise to species with high M_w_ with a UV/Vis emeraldine salt profile. All other samples tested display similar conductivity behavior, presenting slightly higher conductivity when oxidized in the presence of laccase. The higher conductivity obtained for sample (a) also correlates with the crystallinity data which displayed in this case the highest value (Table 2; Rahy and Yang, 2008). The results related with conductivity and XRD confirm the ability of BC to act as a template for the enzymatic polymerization of aniline. Moreover, the enzymatic polymerization in the presence of additives like KHCF and AOT, seems to act synergistically as templates, resulting in high levels of conductivity. Comparing with the control sample, the materials prepared previously by Shim et al. (2019) also using laccase as oxidation catalyst, revealed higher increment, resulting from a higher material deposition onto BC inner and outer surfaces.

#### Coloration Evaluation of BC/PANI

Together with the conductive character conferred by the PANI coated onto BC samples resulting from oxidation, the samples also acquired a different coloration that will depend on the level of oxidation and on the conditions of processing. Table 3 presents the spectral results obtained after aniline oxidation onto BC samples under different processing conditions. The color strength corroborates the previous results reported. As confirmed previously, the additive KHCF, alone or in combination with AOT, in the presence of laccase gave rise to the strongest BC coloration. We have mentioned previously, that by solutions analysis the presence of laccase could be negligible for the oxidation of aniline using KHCF, and looking to these results one may infer that the additives promote oxidation and thereby coloration, however, the catalyst is crucial to achieve strong BC coating and coloration.

**Table 3:** Photographs of BC samples; K/S checksum after polymerization under different conditions, and amount of polymer inside BC.

	**BC sample**	**K/S checksum**	**% polymer inside BC relatively to the amount produced**
Bleached BC	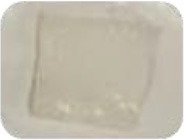	11.42	–
Aniline, LAC	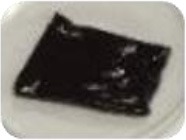	299.02	85
Aniline, LAC, AOT	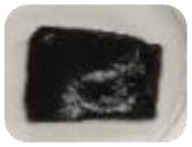	329.08	72
Aniline, No LAC, AOT	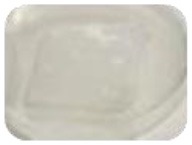	10.74	–
Aniline, LAC, KHCF	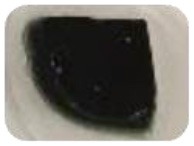	333.01	73
Aniline, No LAC, KHCF	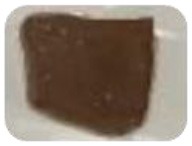	294.07	57
Aniline, LAC, KHCF, AOT	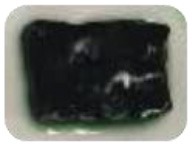	370.97	75
Aniline, No LAC, KHCF, AOT	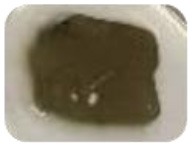	270.05	66

## Conclusions

In this work we study the role of different additives, namely AOT and KHCF, on the aniline polymerization onto bacterial cellulose (BC) supports, using laccase as catalyst. The optimum conditions for the *in situ* polymerization of aniline onto BC were established considering the highest conductivity and coloration results. The synergistic effect between both additives and the enzyme, revealed to be the ideal approach to achieve the best conductive performance. The green PANI obtained by laccase-mediated system in the presence of both additives holds electro-conductive properties displayed in the coated BC membranes, being presented as an advantage for the preparation of materials to be promptly cast onto BC membranes for differentiated applications. The BC coloration is directly proportional to the amount of polymer entrapped which correspond to the highest values of electrical conductivity. The data obtained allowed us to conclude that the oxidation of aniline depends on several factors but the presence of additives acting as templates, guide the reactions toward desired products. The uncertainties related with the evaluation and chemical composition of the reaction products expose procedure fragilities which can be overcome by deeper studies on template-assisted oxidation of aniline.

## Data Availability Statement

All datasets generated for this study are included in the manuscript/Supplementary Files.

## Author Contributions

ES was responsible for experimental and writing details. JN was responsible for HNMR data curation. AC-P was co-supervisor of the work. CS was responsible for experimental details and paper revision. HK was the supervisor of the work.

### Conflict of Interest

The authors declare that the research was conducted in the absence of any commercial or financial relationships that could be construed as a potential conflict of interest.
