# Supplementary material for: Effect of Additives on the in situ Laccase-Catalyzed Polymerization of Aniline Onto Bacterial Cellulose
Source: Front Bioeng Biotechnol. 2019 Oct 17;7:264. doi: 10.3389/fbioe.2019.00264 (PMC6812606; doi:10.3389/fbioe.2019.00264)
Supplement: Supplementary file 1 [file Data_Sheet_1.docx]

**Supporting Information**

**Effect of additives on the *in situ* enzyme-catalyzed polymerization of aniline onto bacterial cellulose**

Euijin Shim, Jennifer Noro, Artur Cavaco-Paulo, Carla Silva*, Hye Rim Kim*

**
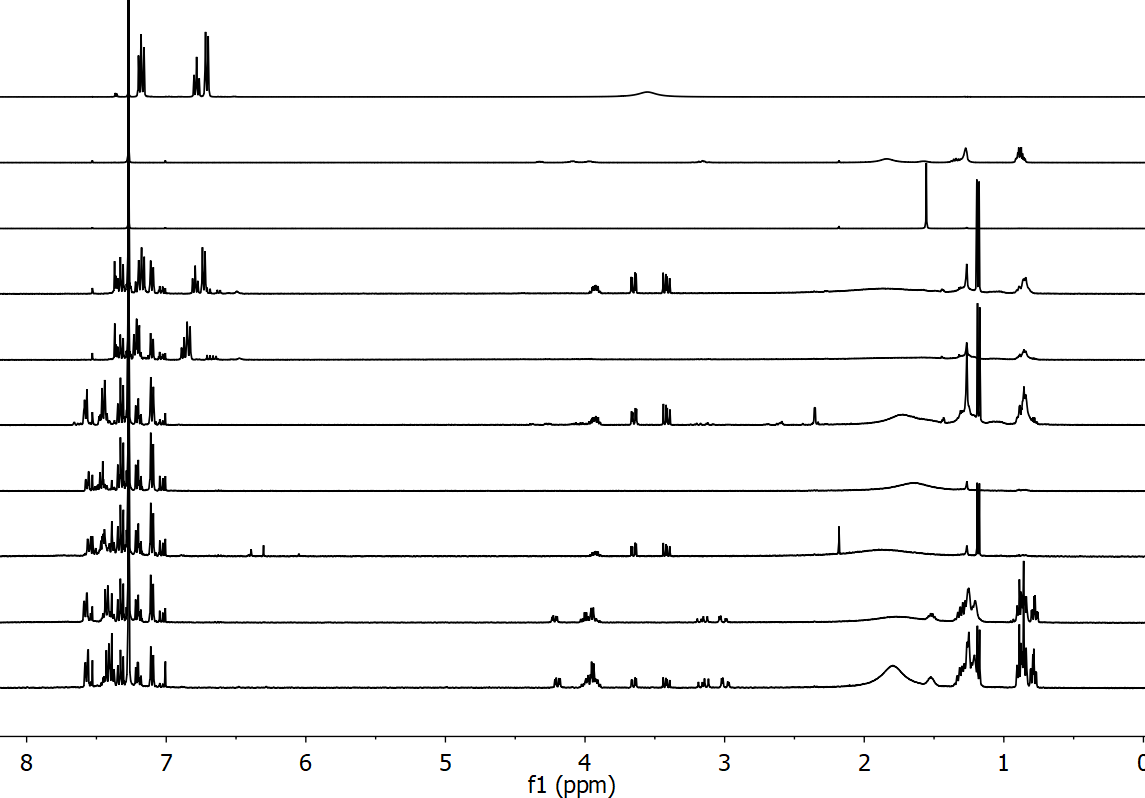
**

a)

b)

a)

b)

c)

d)

e)

f)

g)

h)

i)

j)

c)

d)

e)

f)

g)

h)

i)

j)

**Figure S1:** ^1^H NMR spectra of the products of aniline oxidation by laccase in the presence of additives: a) aniline; b) AOT; c) KHCF; d) aniline + laccase; e) aniline + AOT; f) aniline + AOT + laccase; g) aniline + KHCF; h) aniline + KHCF + laccase; i) aniline + AOT + KHCF; j) aniline + AOT + KHCF + laccase.


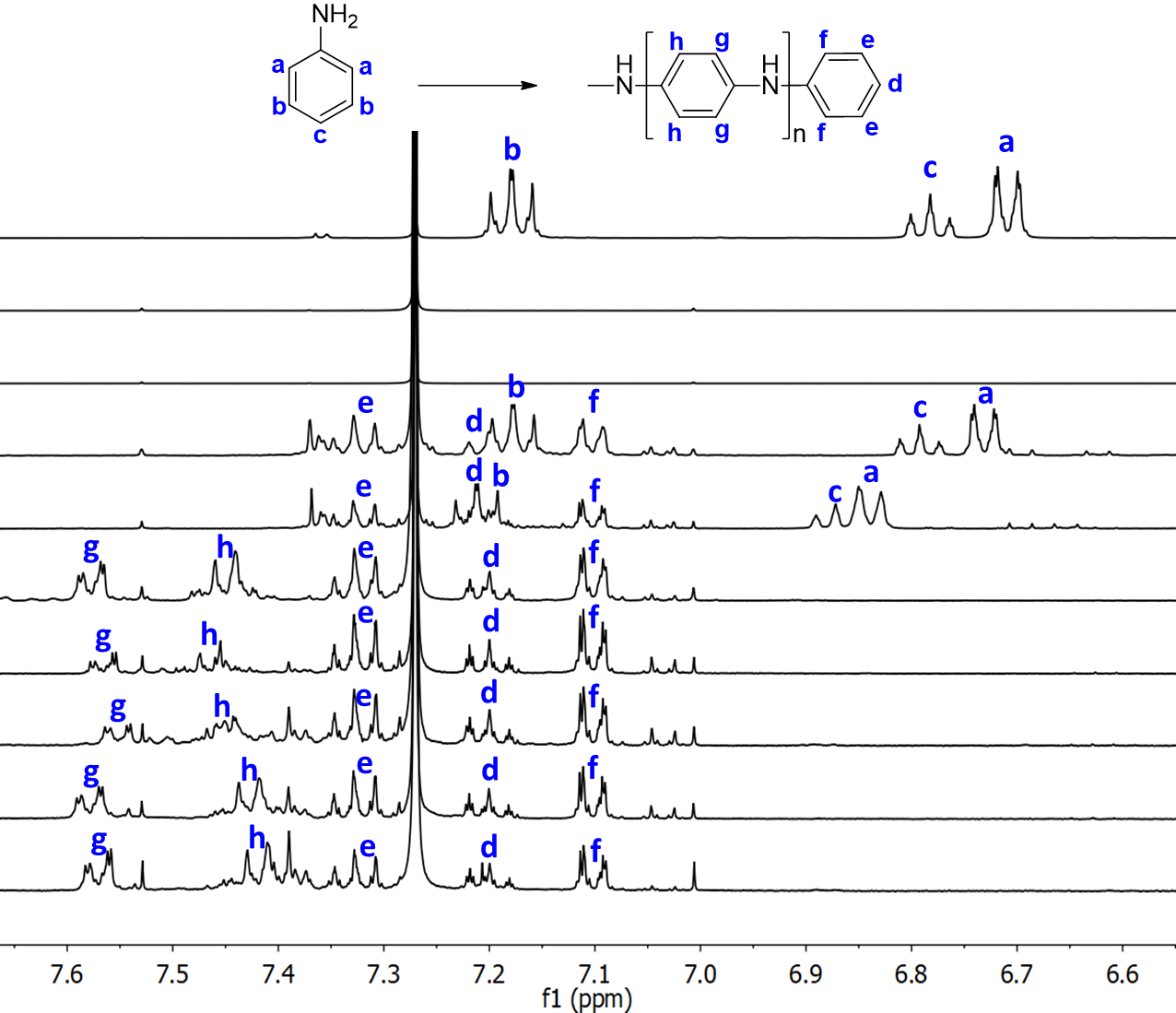


**Figure S2:** ^1^H NMR spectra expansion (6.6 to 7.6 ppm) of the products of aniline oxidation by laccase in the presence of additives: a) aniline; b) AOT; c) KHCF; d) aniline + laccase; e) aniline + AOT; f) aniline + AOT + laccase; g) aniline + KHCF; h) aniline + KHCF + laccase; i) aniline + AOT + KHCF; j) aniline + AOT + KHCF + laccase.


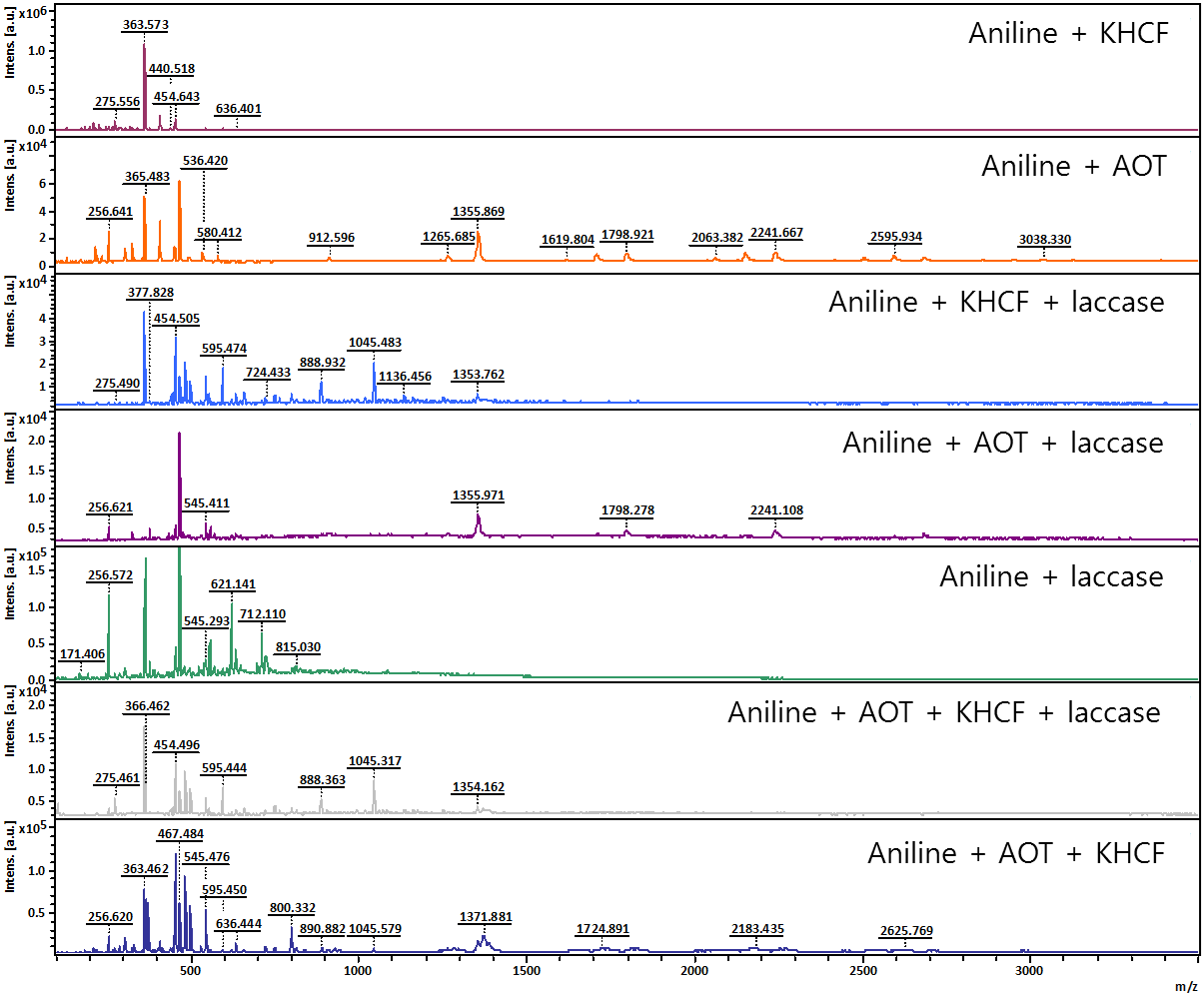


**Figure S3:** MALDI-TOF spectra of the products after aniline polymerization with laccase and additives.


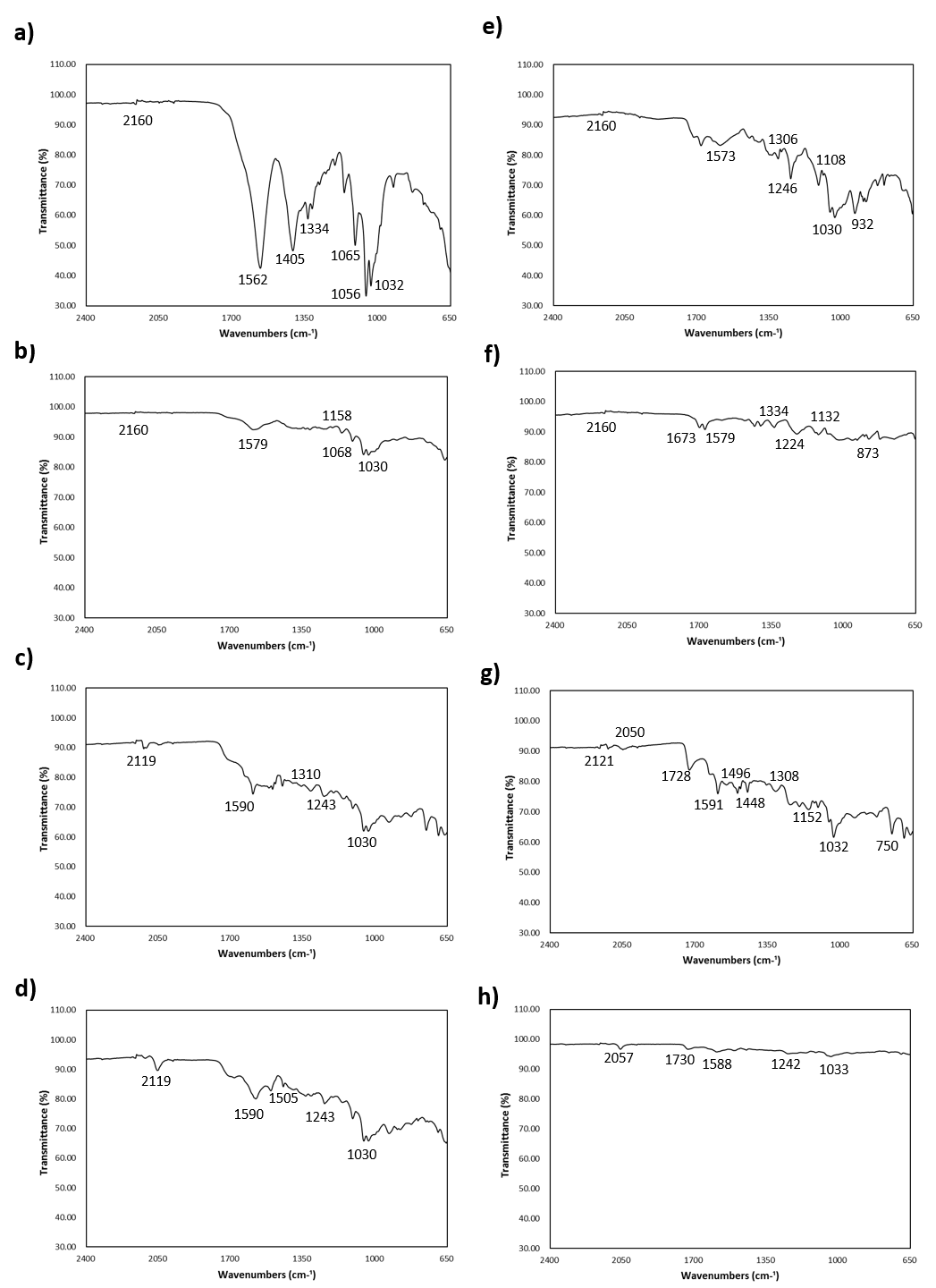


**Figure S4.** FTIR-ATR analysis of BC samples coated with polyaniline resulting from laccase oxidation in the presence of additives: a) BC control; b) BC + aniline + laccase; c) BC + aniline + KHCF + laccase; d) BC + aniline + KHCF; e) BC + aniline + AOT + laccase; f) BC + aniline + AOT; g) BC + aniline + KHCF + AOT + laccase; h) BC + aniline + KHCF + AOT.

| Wavenumber (cm_-1_) | Functionality |
| --- | --- |
| 1,065 | C-O-C stretching vibration |
| 1,448 | C-C stretching of benzenoid rings |
| 1,470 | Stretching of benzoid structure |
| 1,562-1,592 | Stretching of quinoid structure |
| 2,920 | C-H stretching vibration (not shown) |
| 3,332 | -OH stretching vibration (not shown) |

**Table S1:** Characteristic absorption peaks obtained from FTIR spectrum of polyaniline

**
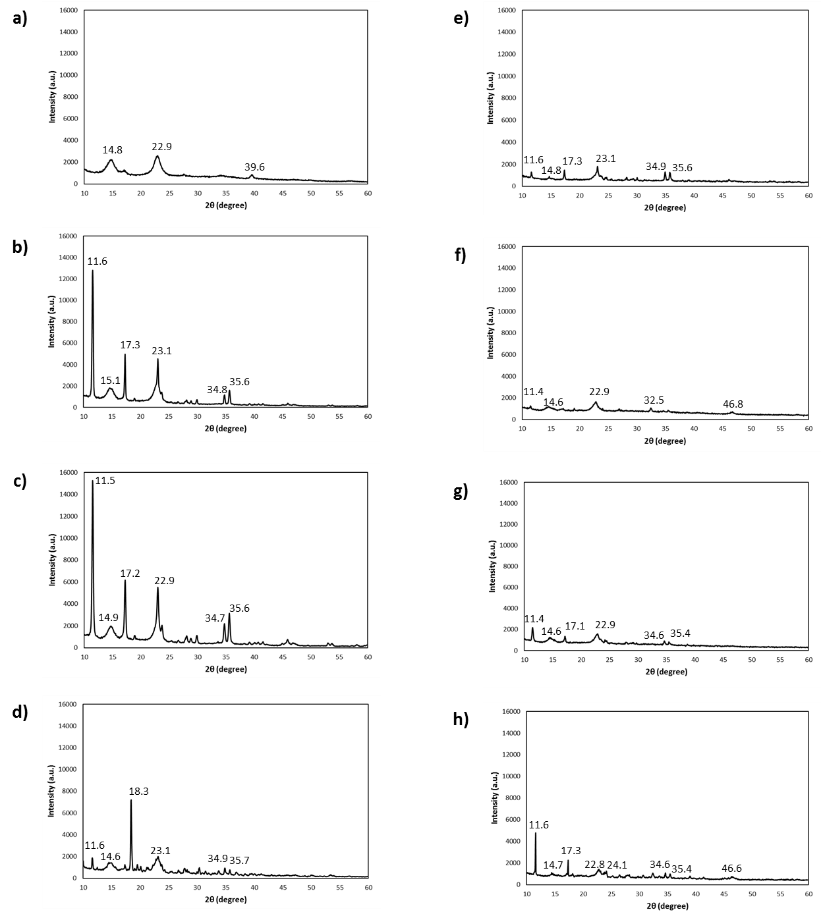
**

**Figure S5:** XRD pattern of BC samples coated with polyaniline synthesized by laccase in the presence of additives: a) BC; b) aniline + laccase; c) aniline + laccase + AOT; d) aniline + without laccase + AOT; e) aniline + laccase + KHCF; f) aniline + without laccase + KHCF; g) aniline + laccase + KHCF + AOT; h) aniline + without laccase + KHCF + AOT.
